# An assessment of cyber security and resilience of the National Digital Health Mission of India

**DOI:** 10.1093/oodh/oqag012

**Published:** 2026-06-07

**Authors:** Suresh Renukappa, Chandrashekar Subbarao, Subashini Suresh, Prashant Pillai, Kieran Fernando, Chandrashekar Rangaswamy, Jayakar Shetty, Rajeev Krishnadas, Tonny Veenith

**Affiliations:** Faculty of Science and Engineering, University of Wolverhampton, Wulfruna Street, Wolverhampton, West Midlands, WV1 1LY, United Kingdom; Department of Critical Care Medicine and Anaesthesia, The Royal Wolverhampton NHS Foundation Trust, New Cross Hospital, Wolverhampton Road, Heath Town, Wolverhampton, West Midlands, WV10 0QP, United Kingdom; Faculty of Science and Engineering, University of Wolverhampton, Wulfruna Street, Wolverhampton, West Midlands, WV1 1LY, United Kingdom; Faculty of Science and Engineering, University of Wolverhampton, Wulfruna Street, Wolverhampton, West Midlands, WV1 1LY, United Kingdom; Faculty of Science and Engineering, University of Wolverhampton, Wulfruna Street, Wolverhampton, West Midlands, WV1 1LY, United Kingdom; Consultant Physician and Associate Medical Director, Midlands Partnership NHS Foundation Trust, St. George's Hospital, Corporation Street, Stafford, Staffordshire, ST16 3SR, United Kingdom; Consultant Anaesthesia, The Royal Wolverhampton NHS Trust, New Cross Hospital, Wolverhampton Road, Heath Town, Wolverhampton, West Midlands, WV10 0QP, United Kingdom; Vice Chancellor, Bangalore University, Jnana Bharathi Campus, Nagarbhavi, Bengaluru, Karnataka 560056, India; Department of Psychiatry, University of Cambridge, Douglas House, 18B Trumpington Road, Cambridge, Cambridgeshire, CB2 8AH, United Kingdom; Faculty of Science and Engineering, University of Wolverhampton, Wulfruna Street, Wolverhampton, West Midlands, WV1 1LY, United Kingdom; Department of Critical Care Medicine and Anaesthesia, The Royal Wolverhampton NHS Foundation Trust, New Cross Hospital, Wolverhampton Road, Heath Town, Wolverhampton, West Midlands, WV10 0QP, United Kingdom

**Keywords:** healthcare services, smart technology, smart healthcare, cybersecurity, cyber resilience, cyber-attacks, digital health, eHealth

## Abstract

The healthcare sector has undergone a transformation driven by the adoption of digital technology. Digital strategies have become the principal mechanism for delivering high-quality, cost-effective healthcare at scale. The Indian Government is also rapidly deploying the Ayushman Bharat Digital Mission (ABDM), launched in September 2021. ABDM is the flagship programme of the Indian Government, which has become the standard platform for healthcare delivery and allied services, including insurance management. The COVID-19 pandemic further accelerated digital adoption, making dependence on digital infrastructure both pervasive and unavoidable. Ensuring cybersecurity and resilience is therefore fundamental to uninterrupted healthcare delivery. This paper examines cyber resilience and cybersecurity within ABDM, with specific reference to its architecture and governing policies. A rigorous assessment of cyber resilience and cybersecurity, encompassing potential threats and attack vectors, is essential to the programme&#×2019;s long-term success. We conclude that ABDM&#×2019;s success is intrinsically linked to robust cybersecurity and resilience. Further work is required to develop a comprehensive framework for ABDM cyber resilience and security that is applicable across all ecosystem partners.

## Introduction

India is undergoing an extensive digital transformation, and healthcare is at the centre of this shift, driven by the Ayushman Bharat Digital Mission (ABDM). According to the UN Population Fund, a growing ageing population, expected to grow to 173 million by 2025 and approximately 240 million by 2050, requires an urgent need for an efficient, robust healthcare delivery system. India is a signatory to Sustainable Development Goal 3 (Good Health and Well-Being) under the 2030 Agenda adopted by the UN General Assembly in 2019. ABDM represents the world’s largest government-funded digital health programme. It is the It provides the backbone for India’s integrated digital health infrastructure, connecting previously fragmented stakeholders across the healthcare ecosystem.

As detailed in the ABDM Strategy (2018), section 3, digital health interventions achieve wider adoption among patients and providers, accelerating progress towards Universal Health Coverage and improving population health outcomes. ABDM aims to improve the quality of care and reduce costs by creating an interoperable digital record system that links patients, providers, and payers. The Indian Government laid out the key principles in the National Health Policy (NHP) (2017), which include universality, citizen-centricity, quality of care, and accountability for performance. This policy vision evolved through the National Health Stack (NHS 2018) into the present ABDM, a digital blueprint encompassing strategic goals, core technical components, and implementation guidelines. The National Digital Health Blueprint (NDHB) outlines the policies and implementation framework for the NHS. The Blueprint also draws on international best practice in digital health strategy.

These foundations motivate a systematic evaluation of ABDM’s approach to cybersecurity and resilience. The importance of cybersecurity in healthcare is well established. The WannaCry ransomware attack of 2017, which crippled large parts of the UK National Health Service, the November 2022 ransomware attack on the All India Institute of Medical Sciences (AIIMS), Delhi, and the subsequent breach of Indian Council of Medical Research (ICMR) data each demonstrate that cyberattacks on health infrastructure are neither hypothetical nor rare. Subbarao et al. (2023) specifically cite ransomware incidents and their impact as evidence to the UK parliament’s security committee. According to Giansanti (2021), the core domains of healthcare cybersecurity are application security, information security, operational security, network security, disaster recovery, resilience, and end-user training, each requiring sector-specific adaptation. Renukappa et al. (2024) argue that all such domains must be addressed collectively in any credible approach to cyber resilience.

Digital healthcare is a highly interconnected ecosystem. Just as multiple digital systems are interconnected, many Internet of Things (IoT) based medical devices have become integral to healthcare delivery. Coventry and Branley (2018) report that 10–15 devices are connected per bed in US hospitals, linking to hospital networks for remote monitoring. However, the expansive use of such medical devices and their interconnectedness introduces a host of cybersecurity vulnerabilities, increasing the attack surface. This proliferation of IoT devices has further heightened concerns about data breaches. The interconnected nature of healthcare networks presents new security challenges, necessitating a deeper understanding of cyber-physical systems (Dogaru 2017). The impact of cyber-attacks can be to debilitate the healthcare network itself or cause security breaches that compromise patients’ private health records. Cybercrime in healthcare encompasses data theft, financial loss, and targeted attacks on medical devices and infrastructure (Perakslis 2014).

The most common cyber threats include phishing, ransomware, denial-of-service attacks, and data breaches. Ransomware is the most prevalent threat and has evolved to the point where Ransomware-as-a-Service (RaaS) platforms enable even low-skilled actors to conduct sophisticated attacks (Renukappa et al. 2024). The healthcare sector, and ABDM specifically, must therefore embed cybersecurity and resilience across its strategy, policy, design, and implementation. Cybersecurity and resilience are inherently multi-dimensional, spanning policy, legislation, technical architecture, implementation, and human behaviour. This paper evaluates cybersecurity and resilience across technical, architectural, and policy dimensions, drawing on ABDM’s publicly available architecture documentation and governing policy instruments.

## Research gap

The literature uses the words smart healthcare, eHealth, and digital health interchangeably. Da Fonseca et al. (2021) define e-health as the application of internet-based technologies to provide healthcare services that improve quality of life and facilitate care delivery. Yaqoob et al. (2022) note that securing distributed healthcare data systems presents significant challenges, particularly where data spans multiple facilities. Where the core infrastructure remains centralized, however, it introduces the risk of single-point failures and systemic data leakage. The Breaches of personal health data carry potentially severe consequences for patients and institutions alike. Given that ABDM’s strategies and policies are still maturing, a systematic evaluation of its cyber resilience and security from both architectural and policy perspectives is timely and necessary. There is little data available on the ABDM portal that would allow us to comprehensively understand ABDM’s security and resilience. A systematic review of cybersecurity and resilience relevant to ABDM yields no relevant results. This is a critical gap. This paper does not perform systems-level threat modelling; rather, it evaluates the general architecture and the policies that directly govern cyber resilience and security.

The literature is similarly limited in its holistic treatment of security and resilience within national digital health infrastructure, a gap this paper seeks to address. Despite the NHP being defined in 2017 and the ABDM being launched in 2021, no dedicated, systematic cybersecurity and resilience framework has been articulated for the programme. India’s national cybersecurity policy, published in March 2023, is generic and sector-agnostic. The healthcare sector requires a specialized security and resilience policy commensurate with the scale and sensitivity of digital health deployment under ABDM.

## Research methodology

This study is grounded in a systematic assessment of cyber resilience and security literature pertinent to the healthcare sector. These findings are applied to ABDM to characterize its approach to cyber resilience and identify gaps in the security information available to different stakeholders. The methodology follows systematic literature review principles to identify, evaluate, and synthesize available evidence in response to a defined research question. The central research question concerns the cybersecurity and cyber resilience considerations relevant to the architectural approach, design, policies, and strategies for digital healthcare in India delivered through ABDM. This query returned no directly relevant results, reflecting the sparse published literature on ABDM, particularly its security and resilience dimensions.

## Multi-disciplinary nature of digital healthcare and the ABDM ecosystem

According to Greaves et al. (2018), digital health is a multi-disciplinary domain spanning patient monitoring, diagnosis, management, prevention, rehabilitation, and long-term care delivery. Therefore, digital technologies are being applied in various ways. Beyond direct clinical care, digital technologies also support appointment management, service discovery, teleconsultation, and patient engagement. One of the core pillars of ABDM is the creation and secure persistence of personal health records (PHRs), and the access to and sharing of these through a consent framework. Digital infrastructure also serves as the connective layer, integrating the diverse players across the ecosystem.

**Figure 1:**
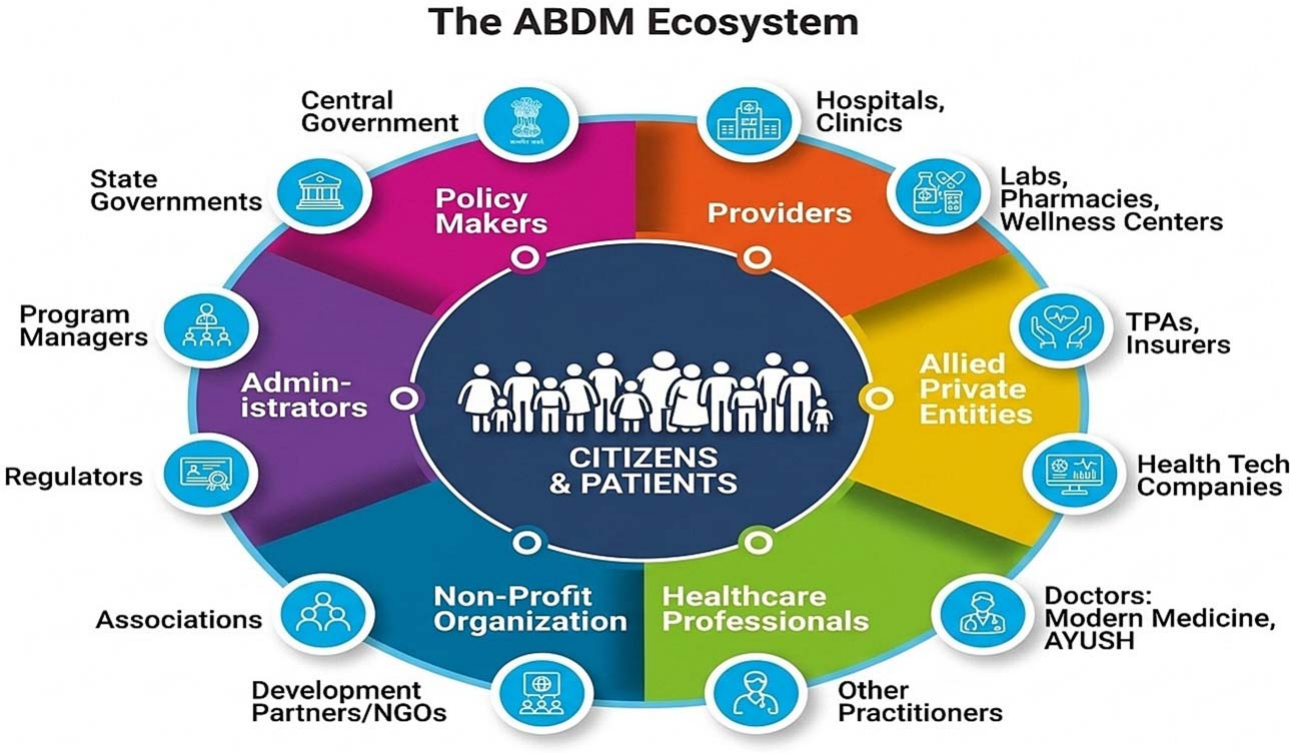
The Ayushman Bharat Digital Mission (ABDM) Digital Health Ecosystem. Source: ABDM (2021).

The ABDM’s ecosystem is complex and comprehensive, and it digitally brings together all the different players and stakeholders, from public sector organizations to healthcare providers, insurance firms, third sector, diagnostic labs, and healthcare service users. With such a diverse range of stakeholders across the National Digital Health Ecosystem (NDHE), ensuring cyber resilience against attacks must be a foremost priority (see Fig. 1). Understanding how ABDM has addressed this challenge through its architecture, policies, and implementation guidelines, and identifying remaining gaps, is therefore essential. Cyber resilience also demands a robust regulatory framework and clearly defined incident response mechanisms.

ABDM creates a digital bridge connecting these real-world entities. Understanding this process is essential for evaluating cybersecurity and resilience across the system. All ecosystem participants have a role in ensuring cyber resilience, with administrators and policymakers setting the framework and service providers implementing it through technical guidelines. With the exception of policymakers and programme managers, every ecosystem partner is directly involved in digital healthcare or allied service delivery, making the cybersecurity challenge correspondingly complex. As diverse ecosystem partners must be brought together, the digital infrastructure will rely on interconnected systems and networks. The interconnected nature of these systems means that a breach in any component can cascade to compromise healthcare delivery across the entire ecosystem. Ensuring cyber resilience across the full digital ecosystem is therefore essential to uninterrupted care delivery, requiring effective mechanisms to isolate affected systems and contain failure propagation.

**Figure 2:**
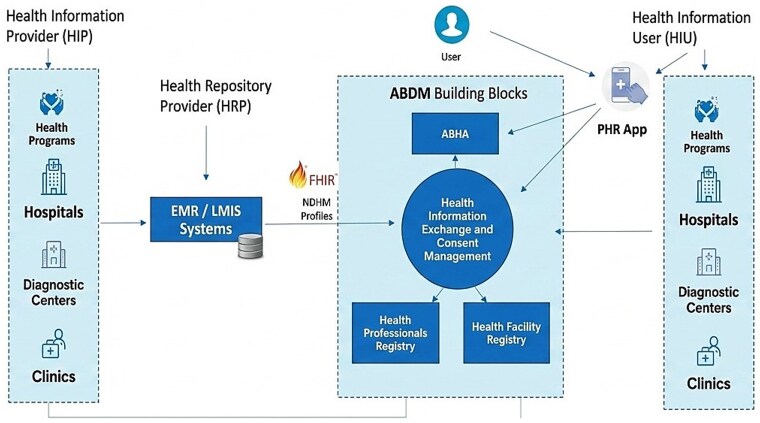
ABDM interaction of functional building blocks with ecosystem partners. ABDM guidelines for HRPs, HIPs, HIUs, section 2 (2022).

Beyond mapping the ecosystem partners, it is equally important to understand how the IT framework integrates them securely and resiliently. To understand this, it is necessary to understand the core functional building blocks of ABDM, which have been conceptualized as a set of ‘digital building blocks’. Each block is seen as a ‘digital public good’ that can be used by any entity in the digital health ecosystem and provides key capabilities—‘ABDM HIU HIP Guidelines (2022)’ (see Fig. 2). These core functional blocks are:

(1) Ayushman Bharat Health Account (ABHA). This is the unique identifier number that a patient is identified with.
(2) Healthcare Professionals Registry (HPR): A central registry is maintained by the National Health Authority (NHA) of all healthcare professionals who will enrol with the HPR. Various recognized medical and nursing councils and paramedical boards will already maintain a professional registry. The HPR will integrate data from all such boards and councils into a central database.
(3) Health Facility Registry (HFR): HFR consists of information for each healthcare facility in the country – hospitals, clinics, diagnostic centres, pharmacies, etc., across all systems of medicine and covering both public and private health facilities.
(4) Health Information Exchange and Consent Manager (HIE-CM): This functional building block plays a crucial role in patients’ data privacy. The HIE-CM enables the exchange of personal health data with the patient’s consent. This data exchange and its security and privacy are guided by the Health Data Management (HDM) Policy issued by NHA. The NHA will run a central HIE-CM, but multiple HIE-CMs are expected to be available in the ecosystem over time.

Once these building blocks are operational, other ecosystem entities integrate with and through them. Real-world partners integrate into the digital ecosystem and are classified by their role in relation to the core system. The most important of these are:

- Health Information Provider (HIP): Any healthcare provider who creates, stores, or distributes health information in the context of providing healthcare-related services to a patient.
- Health Information User (HIU): Any permitted entity that would like to access an individual’s health records with their informed consent is called a HIU.
- Health Repository Provider (HRP): HRPs are software service providers who offer ABDM-compliant software and long-term record storage to hospitals, diagnostic centres, and clinics.
- PHR Apps: PHR Apps are software service providers offering front-end services to individuals, such as creating ABHA, discovering and linking health records, etc.

**Figure 3:**
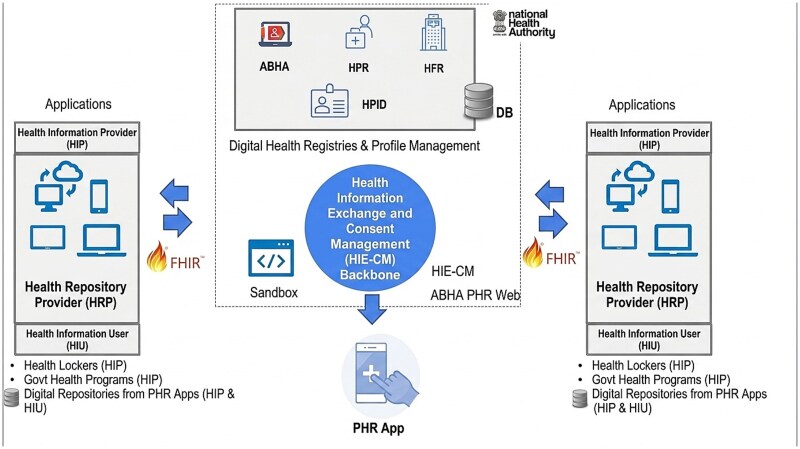
Information Technology Landscape of the Ayushman Bharat Digital Mission (ABDM). ABDM guidelines for HRPs, HIPs, HIUs, section 2 (2022).

The resultant ABDM IT landscape, encompassing the real-world ecosystem, functional building blocks, and their interrelationships, is shown in Fig. 3.

Understanding how the ecosystem’s real-world players get mapped to the digital landscape through the functional building blocks is essential for assessing ABDM in the context of resilience and security. Multiple critical subsystems converge within this landscape. According to Shrivastava et al. (2021), integrating critical subsystems, even small ones, might help adopt more resilient technologies rather than creating a fully functional and resilient integrated system, which is a much more complex and time-consuming process. The IT landscape also delineates ownership: the core National Digital Infrastructure is owned and managed by the NHA, while partner-ecosystem applications operate within it.

## NHA managed ABDM building blocks

The ABDM building blocks are developed, hosted, and managed by the NHA, which serves as their sole custodian. The ABDM building blocks are:

(i) Digital Health Registries & Profile Management.

(a) ABHA.

(b) Healthcare Professional Registry (HPR).

(c) Healthcare Professional ID (HPID).

(d) HFR.

(ii) HIE-CM Backbone:

(a) HIE-CM APIs.

(b) ABHA PHR Web Application.

(iii) Sandbox.

These components constitute the core national digital infrastructure of ABDM and represent the primary focus for security and resilience assurance. The NHA bears direct responsibility for their protection.

## ABDM partner applications

Partner ecosystem managed ABDM Compliant Applications include PHR Apps, HIPs, HIUs, Lab Management Information Systems (LMIS), hospital Management Information Systems (HMIS), and Digital repositories from PHR Apps. They integrate with the critical building blocks of the NHA and together provide end-to-end healthcare services. A key distinction is that end-to-end responsibility for the core building blocks, from design through maintenance, rests entirely with the NHA. This is defined by the NDHM Strategic Control Policy, which aims to retain complete control over the strategic assets of ABDM. Partner ecosystem entities, by contrast, must be guided by clear policies, technical guidelines, and measurable service-level agreements. Integration occurs through well-defined, NHA-certified secure APIs.

Given the interconnected nature of the healthcare industry, cyber resilience cannot be the responsibility of any single entity (WEF 2024). A collaborative, ecosystem-wide approach is essential.

## Cyber resilience and cybersecurity

Cybersecurity and resilience are used hand in hand, with a blurred distinction between the two, though resilience is more commonly the over-arching term. However, having a clear distinction when evaluating these concepts is essential. Resilience can be understood through two related concepts: cyber resilience and cybersecurity. There are various definitions of resilience. According to Haimes (2009), it is the system’s ability to withstand a significant interruption within acceptable degradation parameters and to recover over an acceptable period of time, within reasonable costs and risks. Aven (2017) defined it as the ability to maintain or restore its functionality and performance after a change in the system’s state. Such changes often result from deliberate malicious action. According to Mentges et al. (2023), resilience can also be approached by way of four abilities: (i) to plan and prepare, (ii) to absorb disturbance, (iii) to recover from, and (iv) to adapt to known or unknown threats.

Tzitzili et al. (2023) situate critical infrastructure resilience within this broader definition, characterizing it as the capacity of a system to return to its normal state following a disruptive event (Hosseini et al. 2016). Across these definitions, cyber resilience is distinguished by its emphasis on recovery and resumption after disruption, taking a whole-system perspective. Cybersecurity, by contrast, denotes a system’s capacity to anticipate, detect, and withstand cyber threats and recover from attacks (Patriarca et al. 2022).

While the two concepts overlap, the distinction lies in emphasis. Cybersecurity emphasizes protecting systems from cyber threats rather than recovery post-attack. It involves implementing several measures, such as network security, protection against viruses/trojans, keeping software updated, preventing unauthorized access through secure authorization mechanisms, protecting against data breaches through good security policies, awareness about security, and other preventive measures. Cybersecurity is predominantly preventative, with particular concern for the confidentiality and integrity of data. The definition advanced by the National Academies of Science (NAS) as ‘the ability to prepare and plan for, absorb, recover from, and more successfully adapt to adverse’, has gained broad acceptance across organizations and governance bodies (Larkin et al. 2015).

## Cyber resilience and cybersecurity of the critical infrastructure of ABDM

**Figure 4:**
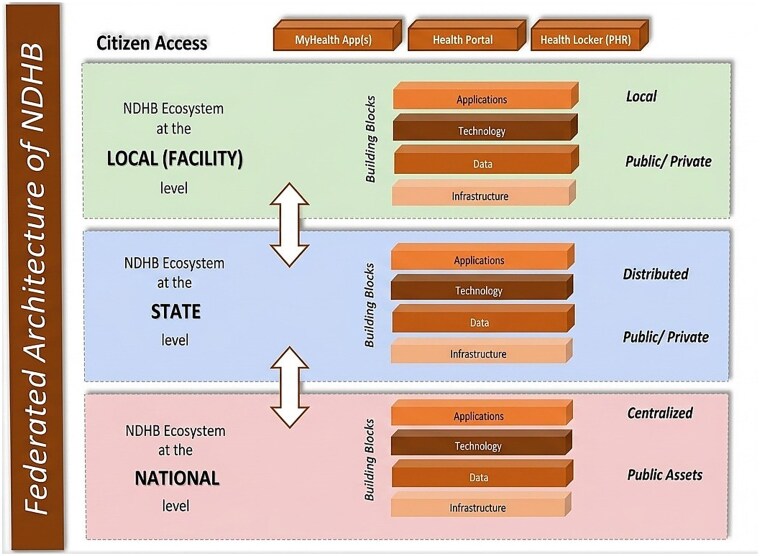
Security-by-Design Principles within the Ayushman Bharat Digital Mission (ABDM) Federated Architecture. Source: ABDM (2019).

ABDM adopts a federated architecture (FA). FA is a pattern in enterprise architecture that allows ‘interoperability and information sharing between semi-autonomous de-centrally organized entities, information technology systems and applications’, according to ABDM Blueprint (2019). Security by design is among the core principles of ABDM (see Fig. 4).

As shown in Fig. 4, the architecture is divided across the national, state, and local facility levels. These layers interact on a need-to-connect basis through well-defined standard APIs. Both central and state government, alongside the private sector, contribute to the digital infrastructure and applications. The IT infrastructure and applications of ABDM are established and maintained in contractual arrangements with the private sector. Strategic assets, however, must remain under the complete control and ownership of the NHA. From a policy perspective, the ‘Strategic Control Policy’ outlined in the ABDM Information Security Policy document enumerates these strategic assets of ABDM to include Software Applications, Databases, Networks, Security, Storage, and Core infrastructure of all the components of the mission, like API gateway, CM, PHR application, and Health ID. These components, therefore, require a robust, dedicated resilience policy. Any vulnerability in these will potentially impact the entire healthcare system, leading to catastrophic consequences.

ABDM’s commitment to data security and privacy is consistently reflected in its policies and FA. FA can support ‘security by design’ and cyber resilience. Federated systems benefit from local autonomy and independent management, improving fault tolerance and enabling isolated remediation of problems, thereby limiting system-wide impact. However, FAs also present challenges. Although the systems are independent, delivering healthcare services will typically require all or many interdependent services to be available. A failure in any single service can disrupt care delivery across the ecosystem; the whole is only as resilient as its weakest component. The critical or strategic infrastructure constitutes many systems that have to integrate. ABDM’s challenge will be to keep all of these constituent and interdependent systems resilient and secure. The distributed, decoupled nature of FA also supports redundancy as an additional resilience benefit. This can help in improving the resilience of the system. Redundancy of both infrastructure and data, where applicable, materially increases resistance to attack.

## Cyber resilience and cybersecurity of the partner ecosystem of ABDM

ABDM’s FA and open API approach are designed to facilitate integration by existing and legacy systems. Requiring wholesale system replacement as a condition of integration would constitute a prohibitive barrier. There are many HMIS, LMIS, and other medical systems that facilities are already using. ABDM addresses this through well-defined APIs that enable incremental, standards-based integration. Integration is nonetheless conditional on demonstrating conformance with defined safeguards. The legacy or existing systems must conform and comply with the ABDM blueprint principles and IndEA (India Enterprise Architecture) principles. Compliance is assessed through a purpose-built evaluation tool prior to integration approval.

The ABDM provides the Sandbox environment to ensure that applications and products are well-tested before integrating into the ABDM ecosystem. This environment enables organizations seeking integration to test compliance with guidelines governing the different building blocks, including HIP, HRP, HIU, and Locker services. The Sandbox hosts all the ABDM building blocks for integration and testing. However, the available documentation reveals that ABDM does not specify resilience-specific requirements or prerequisites for integration. The ‘Secure Application Development Reference Document (NDHM, 2021) provides detailed guidance on secure application and API development. Resilience-specific guidance is absent: questions such as how a system should behave under load, whether APIs implement rate limiting, what scalability thresholds apply, and what the expected behaviour is when a downstream service is unavailable, are not addressed. Nor are there recommendations for resilience design patterns to be applied in the event of inter-system communication failure.

The FA provides the framework for different systems or components to communicate via secure APIs only. The ABDM blueprint lists an Open API-based ecosystem as one of the core principles of ABDM, along with FA. The Government, MeitY (Ministry of Electronics and Information Technology), will notify the Open API policy. Apart from policy and guidelines, security and privacy will be built into the design and development of the APIs. These APIs are also subject to security and privacy audits prior to deployment (ABDM Blueprint 2019).

## Cybersecurity governing policies in ABDM

Data security is central to cybersecurity, and ABDM has consistently accorded it primacy. The Health Data Management (HDM) Policy (2020) addresses this in considerable depth, providing substantive provisions to ensure data security and mitigate privacy and security risks. The NDHB provides the framework underpinning three operational policies:

(1). NDHM Information Security Policy for Internal Ecosystem;

(2). NDHM Information Security Policy for External Ecosystem; and.

(3). NDHM Strategic Control Policy.

Privacy and security challenges span the full ecosystem, from end users to policymakers to implementing organizations (Subbarao et al., 2023). Addressing this requires, as a first step, clear and appropriate policy. The NDHM Information Security Policy for the internal ecosystem specifies safeguards across the NDHE to protect India’s critical digital infrastructure and individual’s health data.

In the ABDM architecture, systems and data are decentralized. Notably, the patient record itself is not centralized. It is stored at the point of care or the nearest physical location. So, the responsibility of data security is also deferred to the data fiduciaries and the data processors. (NDHB, section 2.2.1). The ‘ABDM HIU HIP Guidelines (2022) ‘have specific sections as guidelines for HIUs, HRPs, and HIPs, which also specify the obligations and expectations of each and the integrators. Only the ABHA identifier management system is maintained centrally. The Electronic Health Record (EHR) is a longitudinal, patient-level record distributed across facilities; what is centralized is not the record itself but an index of links to the underlying Electronic Medical Records (EMRs) held at each facility.

As observed, no single overarching cybersecurity or cyber resilience policy governs ABDM. Security and resilience are instead addressed, to varying degrees, across the HDM Policy, the HIP and HIU guidelines, the National Health Stack Strategy, and the ABDM Blueprint. The HDM Policy (2020) is a sector-specific guidance document that operationalizes ‘Security and Privacy by Design’ as its foundational principle for protecting personal digital health data (including Electronic Health Records/EHRs, EMRs, and the ABHA).

For cybersecurity purposes, the HDM Policy treats health data as sensitive personal data and mandates consent-driven, purpose-limited processing with technical and organizational measures to ensure its confidentiality, integrity, and availability (HDM Policy, 2020, Chapter 7).

The policy also mandates security safeguards to be implemented by data fiduciaries, compliance with International Standard IS/ISO/IEC 27001 (Information Security Management System) (HDP, 2020 Chapter 5). It also mandates a comprehensive information security programme, Data Protection Impact Assessments (DPIA), implementing audit trails and breach management procedures (HDM, Chapter 5: Obligations of data fiduciaries), as mandatory obligations of Data fiduciaries.

The Digital Personal Data Protection (DPDPA) Act 2023, operationalized through the DPDP Rules 2025, provides the overarching statutory framework for data privacy and security. The DPDP Act, 2023, is India’s overarching data protection law, which applies to all digital personal data processing. It imposes direct legal accountability on data fiduciaries, including healthcare providers and ABDM ecosystem entities, for cybersecurity and privacy. Health data is treated uniformly as personal data under the Act, without a separate classification.

The HDM Policy is not aspirational guidance: compliance is mandatory for all entities seeking ecosystem participation. Onboarding is conditional on demonstrated adherence, and continued API access is contingent on sustained compliance. This enforcement mechanism, operationalized through the NHA’s onboarding, certification, and access control processes, is a central pillar of cyber resilience at scale within ABDM. Security and privacy obligations are binding, not voluntary. This enforcement architecture determines the practical reach and enforceability of ABDM’s security provisions and must be central to any meaningful resilience assessment. The DPDP Act 2023 and DPDP Rules 2025 reinforce this through statutory obligations that apply independently of ABDM’s onboarding conditions, including mandatory breach notification and incident reporting. Together, ABDM’s policy conditions and India’s data protection law create a layered regulatory architecture that enforces cybersecurity accountability across the ecosystem.

While data security receives substantive attention, other domains of cybersecurity, including network, infrastructure, application, API, and operational security, remain dispersed across multiple documents and are difficult to consolidate into a coherent framework.

India remains governed by the National Cyber Security Policy of 2013 for infrastructure resilience and cybersecurity, despite a fundamental transformation in the scale and complexity of its digital technology landscape since that time. An urgent policy update is overdue.

IoT medical devices are now extensively deployed for clinical monitoring and healthcare delivery. Securing them requires dedicated policy. The 2013 policy does not address IoT, referencing only ‘connected devices’ in passing. An updated policy is overdue. The ‘National Cyber Security Strategy 2020’, submitted by the Data Security Council of India (DSCI) as a consultation document, is a noteworthy step in this direction. It emphasizes sectoral preparedness as a priority. As digitization deepens across all sectors, sector-specific cybersecurity policy will become progressively more necessary. This is particularly true for healthcare, given the sensitivity of the data involved. The UK and the USA, among others, have long had sector-specific healthcare cybersecurity frameworks.

Cybersecurity and resilience within ABDM are enforced not only through architecture but through a controlled onboarding and integration process.

## The ABDM sandbox and the on boarding process to ensure cybersecurity

The **ABDM** Sandbox serves as a controlled testing environment that enables healthcare software developers, EMR/Hospital Information Management System (HIMS) providers, and other integrators to validate compliance with ABDM standards and APIs prior to production deployment. This environment supports integration with core digital building blocks, including the ABHA, HIPs, HIUs, and consent management mechanisms, while ensuring adherence to interoperability standards such as FHIR and secure, consent-based health data exchange.

Applications must undergo a formal exit procedure to transition to the production environment ABDM Sandbox Guidelines (2020). This multi-step certification process, overseen by the NHA, emphasizes both functional correctness and a robust security validation to protect sensitive personal health data in line with the DPDP Act.

Following a Functional and Compliance Testing phase, a Security Assessment and Certification process is undertaken. A mandatory Web Application Security Assessment (WASA) or equivalent audit will be performed by agencies empanelled under Standardization Testing and Quality Certification (STQC) or Indian Computer Emergency Response Team (CERT-IN) (ABDM Sandbox Integration and Exit Process). The assessment rigorously examines the application for vulnerabilities, including Open Worldwide Application Security Project (OWASP) Top 10 risks, secure coding practices, encryption mechanisms, access controls, audit logging, and data protection measures. Successful completion awards a ‘Safe-to-Host’ certificate. This certificate confirms that the application meets the infrastructural and security requirements for hosting within the NDHE and is a prerequisite for production onboarding.

Final approval is granted by the NHA following review. The compliant software is then registered in the ABDM ecosystem, enabling real-world operations such as secure data sharing and integration with the Health Facility Registry (ABDM Sandbox Integration and Exit Process).

This security validation is a cornerstone of the sandbox exit process, reflecting ABDM’s commitment to safeguarding health information within a FA. However, WASA certification is a point-in-time security assessment and does not guarantee operational resilience. Scalability under production load, for example, cannot be validated through sandbox testing alone.

## Digital lockers and cyber resilience

Health Locker services are a functional building block of ABDM, enabling patients to store, access, and share longitudinal PHRs. Health Lockers, operated by ABDM-approved third-party HRPs, allow patients to consolidate health records generated across different episodes of care and multiple facilities into a single, patient-controlled repository. From a cyber resilience perspective, Health Lockers offer two distinct advantages. First, it enables longitudinal patient records to be maintained independently of any single healthcare facility, thereby providing resilience against localized data loss caused by ransomware, system failure, or institutional data breaches. If a hospital’s systems are compromised, the patient’s linked records within the locker remain accessible via other verified HRPs, preserving healthcare continuity. Second, access is governed by the HIE-CM consent framework, constraining unauthorized exfiltration at the architectural level and reducing the risk of large-scale aggregated data breaches.

Health Lockers also introduce resilience risks that remain unaddressed. HRPs are third-party entities whose security posture is assessed at onboarding through WASA, but, as noted, this provides no assurance of post-deployment availability or business continuity. ABDM does not currently specify minimum availability or uptime requirements for HRPs, nor does it mandate disaster recovery or backup obligations. This is a consequential gap: a Health Locker that becomes unavailable following a cyberattack or infrastructure failure could leave patients without access to their records at the point of clinical need. ABDM should extend its resilience requirements for HRPs to include minimum availability standards, mandatory breach notification under the DPDP Act 2023, and data portability provisions to ensure records can be migrated without loss to an alternative provider. Addressing these gaps would substantially strengthen the resilience contribution of Health Lockers across the ecosystem.

## Conclusions and recommendations

The cyber threat to healthcare continues to grow in scale and sophistication. The World Economic Forum reports more than 1000 cyberattacks on healthcare organizations per week globally, making healthcare the third most targeted sector in 2023 (WEF 2023). The need for robust cybersecurity and resilience mechanisms is beyond dispute. This paper has examined key architectural and policy considerations bearing on ABDM’s resilience and security. A central finding is that cyber resilience in ABDM is not explicitly articulated as a unifying concept. Data security and privacy receive substantially greater policy attention and articulation. The ecosystem assessment also confirms that cyber resilience demands genuine collaboration between government and private sector partners. As the WEF (2024) observes, resilience is the defining challenge. This requires all actors to understand their responsibilities clearly, and a standardized mechanism for assessing and measuring cybersecurity and resilience. Given the number of data fiduciaries, registries, and private entities involved, security and resilience obligations must be driven by clear policy and enforceable compliance mechanisms.

ABDM should develop and publish a dedicated assessment framework for security and resilience, modelled on the Cyber Assessment Framework (CAF) of the UK National Cyber Security Centre (NCSC). Such a framework, customized to the ABDM context, should become a condition of ecosystem integration. Renukappa et al. (2024) have noted the value of the CAF in tracking progress towards sector-wide cyber resilience. ABDM’s existing API compliance assessment tool for legacy systems should be extended to all integrating systems and made available as a self-assessment instrument, enabling partners to evaluate their position before formal integration. To clarify the rationale for this recommendation, it is instructive to compare the CAF’s structure with ABDM’s current approach to cyber resilience governance. The UK CAF is a structured, outcome-based framework organized around four top-level objectives: (i) Managing security risk; (ii) Protecting against cyberattack; (iii) Detecting cybersecurity events; and (iv) Minimizing the impact of incidents, underpinned by fourteen contributing outcomes and measurable indicators of good practice (CAF v4, NCSC). It is designed for Operators of Essential Services and is adaptable to complex national digital infrastructure such as ABDM. By contrast, ABDM’s current cyber governance model is fragmented across multiple policy documents: the NDHM Information Security Policy, the Strategic Control Policy, the HDM Policy, and the Secure Application Development Reference Document address different aspects of security but do not cohere into a unified, measurable resilience framework. The focus is predominantly on data security and privacy obligations for data fiduciaries, with limited articulation of availability, continuity, and incident recovery requirements for the ecosystem as a whole. The WASA-based onboarding certification, while valuable, is a point-in-time assessment of application security rather than a continuous resilience assurance mechanism. A CAF-modelled framework would address these gaps by establishing clear, measurable outcomes for all ABDM stakeholders, including NHA, HRPs, HIPs, HIUs, and PHR App providers, covering protection, detection, and recovery. The CAF approach also supports proportionality, enabling different tiers of the ABDM ecosystem to be assessed against outcomes commensurate with their criticality, rather than applying uniform requirements across all partners. Adopting such a framework would provide a credible, internationally benchmarked mechanism for ongoing resilience assurance, complementary to the existing WASA certification process.

A dedicated framework for the NHA-owned critical national infrastructure of ABDM is also warranted. Rehak et al. (2019) demonstrate the value of structured methodologies for measuring resilience in critical infrastructure, and their Critical Infrastructure Elements Resilience Assessment (CIERA) methodology offers a directly applicable model. ABDM should consider formally adopting a CIERA-equivalent approach for its core infrastructure. Human factors remain central to security: all users working with healthcare provider systems must receive substantive security training. Training programmes must be regularly updated and delivered at intervals sufficient to address an evolving threat landscape. A robust, people-centric approach to cyber defence is essential, given that the majority of malware infections originate through human error or social engineering. ABDM should mandate security awareness and training standards across all participating organizations.

Furthermore, with the advent of AI and its application in almost every sphere, AI-based cyberattacks and deepfakes pose a new level of risk that healthcare organizations and their technology partners must be prepared to confront this threat. This threat from AI is not yet fully understood, as AI is itself nascent. Even the policy paper on cybersecurity has very little reference to AI-powered threats or how AI could be used to mitigate and engineer a danger.

The following specific recommendations are drawn from this assessment. People: Mandatory training in information governance, phishing recognition, and safe digital practices is essential. Regular security awareness programmes, adapted to the Indian healthcare context, should be a standard requirement for all system users. Network: Network security standards governing protocols, encryption, and access controls, as established in the UK and USA, provide directly applicable models that India should adapt for ABDM. Software: Software vulnerability management, including timely patch cycles and dependency auditing, must be formalized as an ABDM ecosystem requirement. Tools: Data Loss Prevention (DLP) and Information Protection tools should be deployed across the ecosystem to mitigate inadvertent data leakage.

## Study funding and APC funding

This research received no specific grant from any funding agency in the public, commercial, or not-for-profit sectors.

## Data Availability

There are no new data associated with this article.

## References

[ref1] ABDM . Ayushman Bharat Digital Mission, Government of India. New Delhi, India: NITI Aayog, Government of India, 2021. https://abdm.gov.in/home (28 February 2024, date last accessed).

[ref2] ABDM HIU HIP Guidelines 2022 Guidelines for HIPs and HIUs, HRPS and PHR Apps, Ayushman Bharat Digital Mission, Government of India, Policies and Guidelines document. https://abdm.gov.in/strapicms/uploads/HIP_HIU_Guidelines_f85df336ec.pdf (23 February 2026, date last accessed).

[ref3] ABDM National Digital Health Blueprint . Ayushman Bharat Digital Mission, Government of India, Strategy document. New Delhi, India: NITI Aayog, Government of India, 2019. https://abdm.gov.in:8081/uploads/ndhb_1_56ec695bc8.pdf (21 February 2026, date last accessed).

[ref4] ABDM Strategy . Ayushman Bharat Digital Mission, Government of India, Strategy document. New Delhi, India: NITI Aayog, Government of India, 2018. https://abdm.gov.in/strapicms/uploads/NHS_Strategy_and_Approach_1_89e2dd8f87.pdf (28 February 2026, date last accessed).

[ref6] ABDM Sandbox Guidelines . NDHM Sandbox Enabling Framework, 2020. https://abdm.gov.in/strapicms/uploads/sandbox_guidelines_b39bcce23e.pdf (8 April 2026, date last accessed).

[ref7] Aven T . How some types of risk assessments can support resilience analysis and management. *Reliability Engineering & System Safety* 2017;167:536–43. 10.1016/j.ress.2017.07.005

[ref8] Coventry L, Branley D . Cybersecurity in healthcare: A narrative review of trends, threats and ways forward. Maturitas. 2018;113:48–52. 10.1016/j.maturitas.2018.04.008 29903648

[ref11] Dogaru DI, Dumitrache I. Cyber security in healthcare networks. In: E-Health and Bioengineering Conference (EHB). Sinaia, Romania: Institute of Electrical and Electronics Engineers, 2017, 414–7. 10.1109/EHB.2017.7995449

[ref12] da Fonseca MH, Kovaleski F, Picinin CT et al. E-health practices and technologies: a systematic review from 2014 to 2019. *Healthcare.* 2021;9:1192. 10.3390/healthcare9091192 34574966 PMC8470487

[ref13] Rehak D, Senovsky P, Hromada M et al. Complex approach to assessing resilience of critical infrastructure elements. *Int J Crit Infrastruct Prot* 2019;25:125–38. 10.1016/j.ijcip.2019.03.003

[ref15] DPDPA . The Digital Personal Data Protection Act, 2023. New Delhi: Ministry of Electronics and Information Technology, Government of India, 2023. https://www.meity.gov.in/static/uploads/2024/06/2bf1f0e9f04e6fb4f8fef35e82c42aa5.pdf

[ref16] DPDP Rules 2025. Digital Personal Data Protection Rules, 2025. Ministry of Electronics and Information Technology, Government of India. Gazette Notification No. G.S.R. 846(E), 13 November 2025. https://www.meity.gov.in/static/uploads/2025/11/53450e6e5dc0bfa85ebd78686cadad39.pdf (9 April 2026, date last accessed).

[ref17] Greaves F, Joshi I, Campbell M et al. What is an appropriate level of evidence for a digital health intervention? *Lancet* 2018;392:2665–7. 10.1016/S0140-6736(18)33129-5 30545779

[ref18] Georgia A. Tzitzili, Stergiani Spyrou, Agapios N. (2023). An Analysis of Resilience and Risk Assessment in Digital Healthcare Systems Platis Proceedings of the 33rd European Safety and Reliability Conference (ESREL 2023). 10.3850/978-981-18-8071-1_P420-cd

[ref19] Giansanti D . Cybersecurity and the digital-health: the challenge of this millennium. *Healthcare (Basel)* 2021;9:62. 10.3390/healthcare9010062 33440612 PMC7827661

[ref20] Haimes YY . On the definition of resilience in systems. *Risk Anal* 2009;29:498–501. 10.1111/j.1539-6924.2009.01216.x 19335545

[ref21] HDM, Health Data Management policy . Ayushman Bharat Digital Mission, Government of India, Strategy document. New Delhi, India: National Health Authority, Government of India, 2020. https://abdm.gov.in/publications/policies_regulations/health_data_management_policy (21 February 2026, date last accessed).

[ref21a] HDP, Health Data Management Policy . New Delhi, India: National Health Authority, Government of India, 2020. Available at: https://abdm.gov.in/strapicms/uploads/health_management_policy_bac9429a79.pdf

[ref22] Hosseini S, Barker K, Ramirez-Marquez JE. A review of definitions and measures of system resilience. *Reliability Engineering & System Safety* 2016;145:47–61. 10.1016/j.ress.2015.08.006

[ref26] Larkin S, Fox-Lent C, Eisenberg DA et al. Benchmarking agency and organisational practices in resilience decision making. *Environ Syst Decis* 2015;35:185–95. 10.1007/s10669-015-9554-5

[ref22a] Mentges A, Halekotte L, Schneider M et al. A resilience glossary shaped by context: Reviewing resilience-related terms for critical infrastructures. International Journal of Disaster Risk Reduction. 2023;96:103893. Available at: 10.1016/j.ijdrr.2023.103893

[ref28] National Health Policy . 2017. https://main.mohfw.gov.in/sites/default/files/9147562941489753121.pdf (28 February 2024, date last accessed).

[ref29] National Health Stack . The National Health Stack, Strategy and Approach, New Delhi, India: NITI Aayog (National Institution for Transforming India), Government of India, 2018. https://abdm.gov.in:8081/uploads/NHS_Strategy_and_Approach_1_89e2dd8f87.pdf (28 February 2024, date last accessed).

[ref30] NDHM Secure Application Development- Reference document 2021. https://sandbox.abdm.gov.in/documents/NDHM_Secure_Application_Development-Reference_Document.pdf (28 February 2024, date last accessed).

[ref32] Patriarca R, Simone F, Di Gravio G. Modelling cyber resilience in a water treatment and distribution system. *Reliability Engineering & System Safety* 2022;226:108653. 10.1016/j.ress.2022.108653

[ref33] Perakslis ED . Cybersecurity in health care. *N Engl J Med* 2014;371:395–7. 10.1056/NEJMp1404358 25075831

[ref35] Renukappa S, Subbarao CK, Suresh S et al. Securing the critical national healthcare infrastructure from cyber attacks, written evidence CYB0010, cyber resilience of the UK's critical national infrastructure. London, United Kingdom: UK Parliament, 2024. https://committees.parliament.uk/writtenevidence/126352/pdf/ (28 February 2024, date last accessed).

[ref35a] Renukappa S, Mudiyi P, Suresh S et al. Evaluation of challenges for adoption of smart healthcare strategies. Smart Health 2024:26. 10.1016/j.smhl.2022.100330

[ref36] Shrivastava U, Hazarika B, Rea A. Restoring clinical information system operations post data disaster: the role of IT investment, integration and interoperability. *Ind Manag Data Syst* 2021;121:2672–96. 10.1108/IMDS-03-2021-0128

[ref37] Subbarao CK, Renukappa S, Suresh S et al. Ransomware Cyber Attacks in the Healthcare sector, Written evidence RAN0013. National Security Strategy (Joint Committee). London, United Kingdom: UK Parliament, 2023. https://committees.parliament.uk/work/7017/ransomware/publications/written-evidence/?page=2 (28 February 2024, date last accessed).

[ref42] The National Cyber Security Strategy . Data Security Council of India, 2020. https://www.dsci.in/files/content/knowledge-centre/2023/National-Cyber-Security-Strategy-2020-DSCI-submission.pdf

[ref38] World Economic Forum . Healthcare Cyber Attacks are on the Rise: Here's Why Zero Trust will Prevent Care Disruptions. Geneva, Switzerland: World Economic Forum, 2023. https://www.weforum.org/agenda/2023/05/cyber-attacks-on-healthcare-rise-zero-trust/ (20 February 2024, date last accessed).

[ref10] World Economic Forum (WEF) . Cyber resilience is the key. 2024. https://www.weforum.org/agenda/2024/02/healthcare-pays-the-highest-price-of-any-sector-for-cyberattacks-that-why-cyber-resilience-is-key/ (20 February 2026, date last accessed).

[ref39] Yaqoob I, Salah K, Jayaraman R et al. Blockchain for healthcare data management: opportunities, challenges, and future recommendations. *Neural Comput & Applic* 2022;34:11475–90. 10.1007/s00521-020-05519-w

